# Dataset of the livability performance of the city of Birmingham, UK, as measured by its citizen wellbeing, resource security, resource efficiency and carbon emissions

**DOI:** 10.1016/j.dib.2017.10.004

**Published:** 2017-10-13

**Authors:** Joanne M. Leach, Susan E. Lee, Christopher T. Boyko, Claire J. Coulton, Rachel Cooper, Nicholas Smith, Hélène Joffe, Milena Büchs, James D. Hale, Jonathan P. Sadler, Peter A. Braithwaite, Luke S. Blunden, Valeria De Laurentiis, Dexter V.L. Hunt, AbuBakr S. Bahaj, Katie Barnes, Christopher J. Bouch, Leonidas Bourikas, Marianna Cavada, Andrew Chilvers, Stephen J. Clune, Brian Collins, Ellie Cosgrave, Nick Dunn, Jane Falkingham, Patrick James, Corina Kwami, Martin Locret-Collet, Francesca Medda, Adriana Ortegon, Serena Pollastri, Cosmin Popan, Katerina Psarikidou, Nick Tyler, John Urry, Yue Wu, Victoria Zeeb, Chris D.F. Rogers

**Affiliations:** a University of Birmingham, Department of Civil Engineering, School of Engineering, Edgbaston, Birmingham B15 2TT, UK; b Lancaster University, Imagination Lancaster, Bailrigg, Lancaster LA1 4YW, UK; c University College London, Department of Psychology and Language Sciences, 26 Bedford Way, London WC1H 0AP, UK; d University of Southampton, Department of Sociology, Social Policy & Criminology, University Road, Southampton SO17 1BJ, UK; e University of Birmingham, Department of Geography Earth and Environmental Sciences, Edgbaston, Birmingham B15 2TT, UK; f University of Southampton, Energy and Climate Change Division, Engineering and the Environment, University Road, Southampton SO17 1BJ, UK; g University College London, Department of Science Technology Engineering and Public Policy, Gower Street, London WC1E 6BT, UK; h University of Southampton, Department of Social Statistics & Demography, University Road, Southampton SO17 1BJ, UK; i University College London, Department of Civil Environmental and Geomatic Engineering, Gower Street, London WC1E 6BT, UK; j University College London, Quantitative & Applied Spatial Economic Research Laboratory, Gower Street, London WC1E 6BT, UK; k Lancaster University, Department of Sociology, Bailrigg, Lancaster LA1 4YW, UK

## Abstract

This data article presents the UK City LIFE_1_ data set for the city of Birmingham, UK. UK City LIFE_1_ is a new, comprehensive and holistic method for measuring the livable sustainability performance of UK cities. The Birmingham data set comprises 346 indicators structured simultaneously (1) within a four-tier, outcome-based framework in order to aid in their interpretation (e.g., promote healthy living and healthy long lives, minimize energy use, uncouple economic vitality from CO2 emissions) and (2) thematically in order to complement government and disciplinary siloes (e.g., health, energy, economy, climate change). Birmingham data for the indicators are presented within an Excel spreadsheet with their type, units, geographic area, year, source, link to secondary data files, data collection method, data availability and any relevant calculations and notes. This paper provides a detailed description of UK city LIFE_1_ in order to enable comparable data sets to be produced for other UK cities. The Birmingham data set is made publically available at http://epapers.bham.ac.uk/3040/ to facilitate this and to enable further analyses. The UK City LIFE_1_ Birmingham data set has been used to understand what is known and what is not known about the livable sustainability performance of the city and to inform how Birmingham City Council can take action now to improve its understanding and its performance into the future (see “Improving city-scale measures of livable sustainability: A study of urban measurement and assessment through application to the city of Birmingham, UK” Leach et al. [2]).

**Specifications Table**

**Table:** 

Subject area	*Urban studies and sustainability*
More specific subject area	*Data analytics for understanding urban livable sustainability*
Type of data	*Spreadsheet*
How data was acquired	*Secondary data were downloaded from various sources (specified in the spreadsheet). Primary data were obtained via various surveys (specified in the spreadsheet).*
Data format	*Raw, Filtered, Analyzed*
Experimental factors	*Indicators were selected from multiple sources based upon their relevance to UK urban livable sustainability: human and societal wellbeing, resource security and efficiency, and carbon emissions.*
Experimental features	*Indicators were classified by outcome and theme for the purpose of aiding data interpretation.*
Data source location	*Within the political boundary of the city of Birmingham, UK*
Data accessibility	*The UK City LIFE*_*1*_*Birmingham data set is free and publically available to download from*http://epapers.bham.ac.uk/3040/
Related research article	Leach JM, Lee SE, Hunt DVL, Rogers CDF. Improving city-scale measures of livable sustainability: A study of urban measurement and assessment through application to the city of Birmingham, UK. Cities. 2017 71:80-87.

**Value of the data**

• This data set captures the livable sustainability performance of the city of Birmingham, UK. The format and information contained within the spreadsheet are designed to enable others to collect livable sustainability data for other UK cities and make possible comparisons across cities. Should data for enough UK cities be collected then statistical analyses across the cities would become possible (e.g., factor analysis), providing unique insights into the interconnected nature of the indicators and how UK cities perform.
• The data set describes Birmingham, UK's livable sustainability performance as a snapshot (i.e., it does not include longitudinal data). Therefore, there is an opportunity to augment the data set by incorporating longitudinal data.
• The data set is not constrained by data type or scale, requiring only that the data be representative of the entire city of Birmingham. This limits statistical analyses, but creates opportunities for other forms of analyses and in particular for innovative data visualization.
• Expanded analyses of the data are possible through comparison with sub-city-scale areas of Birmingham (e.g., neighborhoods), subject to the collection of neighborhood-scale data.
• The UK city LIFE_1_ format can be tailed to other urban contexts, such as cities outwith the UK.

## 1 Data

The UK City LIFE_1_ (UK City Livable-sustainability Indicator Framework Edition 1) Birmingham data set presents the livable sustainability performance of the city of Birmingham, UK presented in a multi-tab spreadsheet containing 346 indicators.

**Fig. 1:**
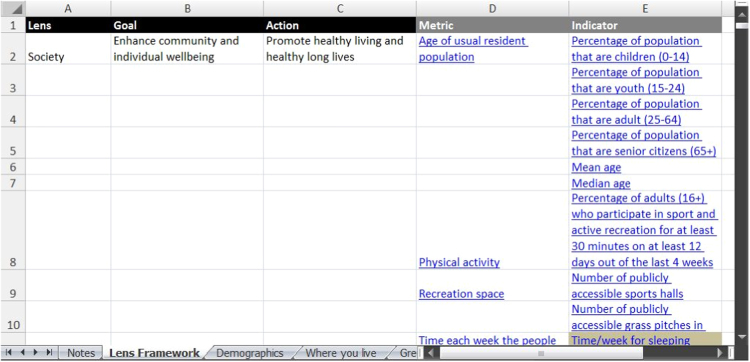
'Lens Framework' spreadsheet tab (excerpt).

The indicators are organized in two ways. The first is within a four-tier, outcome-focused framework (‘Lens Framework’). The framework links the least granular of desired outcomes (the four lenses of sustainability: society, environment, economy and governance) to related goals (e.g., enhancing community and individual wellbeing, enhancing biodiversity and ecosystem services) and actions (e.g., promoting healthy living and healthy long lives, minimizing the impact of urban density on biodiversity), finally to the granularity of metrics and indicators (e.g., healthy life expectancy, quality of waterways) [1]. The Lens Framework can be found on the second tab of the spreadsheet (see Fig. 1). The metrics and indicators are hyperlinked to their full descriptions, which are contained within the spreadsheet's tabs.

**Fig. 2:**
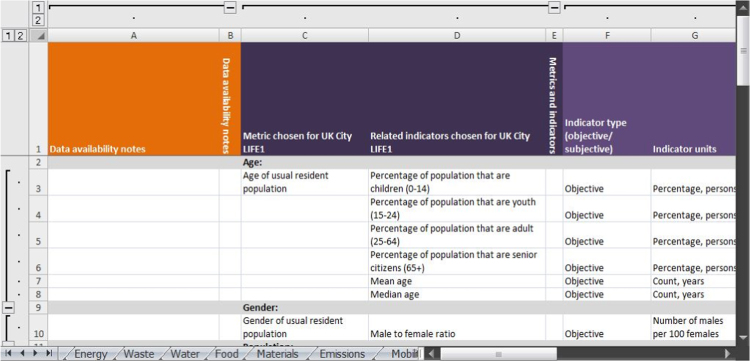
Themed spreadsheet tabs (excerpt).

The second way the indicators are organized is by theme. The themes have been selected to complement government and disciplinary siloes (e.g., health, energy, economy, climate change). Tabs three to 24 within the spreadsheet contain the indicators that correspond with the themes (see Fig. 2). Birmingham data for the indicators are presented on each tab, are grouped by metric and include indicator type, units, geographic area, year, source, link to secondary data files, data collection method, data availability and any relevant calculations and notes.

## 2 Experimental design, materials and methods

UK City LIFE_1_ is a unique and bespoke city performance measurement and assessment method designed to provide a comprehensive and holistic account of a UK city's livable sustainability. It includes subjective and objective measures and is not restricted by data type (e.g., quantitative, qualitative, categorical, index, etc.). UK City LIFE_1_ has been used to measure the livable sustainability performance of Birmingham, UK and the arising data set is freely and publically available at http://epapers.bham.ac.uk/3040/. A description and critique of the development of UK City LIFE_1_ is available from Leach et al. [2].

In order to be included in the UK City LIFE_1_ Birmingham data set, data were required to be representative of the city of Birmingham, as defined by its political boundary, but did not necessarily have to have sub-city scale components. Data for Birmingham were collected as a first preference for 2011 (given the prevalence of 2011 Census data), as a second preference for the least recent year after 2011, and as a third preference for the most recent year prior to 2011 [2]. The data set does not contain longitudinal data. The data set is a combination of data from secondary sources and primary sources, with data collection methods and calculations included in the spreadsheet on an indicator-by-indicator basis. Secondary data sources were the preference and sources were selected for their reputation for providing high quality data. In some cases it was deemed necessary for less-robust data to be included as having no data would unnecessarily compromise the balance of the data set. Where no secondary data sources existed or were easily obtainable (e.g., restricted access) and where it was not feasible to conduct primary data collection, indicator values were marked as null. As a result of utilizing data from multiple sources, there are varying cohort sizes, data collection methods and timestamps across the indicators.

## Funding

The authors gratefully acknowledge the financial support of the UK Engineering and Physical Sciences Research Council (EPSRC) under grant EP/J017698/1: Transforming the Engineering of Cities to Deliver Societal and Planetary Wellbeing.
