# Supplementary material for: Dataset of the livability performance of the city of Birmingham, UK, as measured by its citizen wellbeing, resource security, resource efficiency and carbon emissions
Source: Data Brief. 2017 Oct 13;15:691–5. doi: 10.1016/j.dib.2017.10.004 (PMC5671466; doi:10.1016/j.dib.2017.10.004)
Supplement: Supplementary file 1 — Supplementary material [file mmc1.docx]

Conflicts of interest declaration

We wish to confirm that there are no known conflicts of interest associated with this publication and there has been no significant financial support for this work that could have influenced its outcome.

We confirm that the manuscript has been read and approved by all named authors and that there are no other persons who satisfied the criteria for authorship but are not listed. We further confirm that the order of authors listed in the manuscript has been approved by all of us.

We confirm that we have given due consideration to the protection of intellectual property associated with this work and that there are no impediments to publication, including the timing of publication, with respect to intellectual property. In so doing we confirm that we have followed the regulations of our institutions concerning intellectual property.

We understand that the Corresponding Author is the sole contact for the Editorial process (including Editorial Manager and direct communications with the office). She is responsible for communicating with the other authors about progress, submissions of revisions and final approval of proofs. We confirm that we have provided a current, correct email address which is accessible by the Corresponding Author.

Signed by all authors as follows, on the 8^th^ of September 2017:

Joanne M Leach – corresponding author

Susan E Lee

Christopher T Boyko

Claire J Coulton

Rachel Cooper

Nicholas Smith

Hélène Joffe

Milena Büchs

James D Hale

Jonathan P Sadler

Peter A Braithwaite

Luke S Blunden

Valeria De Laurentiis

Dexter V L Hunt

AbuBakr S Bahaj

Katie Barnes

Christopher J Bouch

Leonidas Bourikas

Marianna Cavada

Andrew Chilvers

Stephen J Clune

Brian Collins

Ellie Cosgrave

Nick Dunn

Jane Falkingham

Patrick James

Corina Kwami

Martin Locret-Collet

Francesca Medda

Adriana Ortegon

Serena Pollastri

Cosmin Popan

Katerina Psarikidou

Nick Tyler

John Urry

Yue Wu

Victoria Zeeb

Chris D F Rogers
